# Molecular Mechanisms Regulating the Dendritic Development of Newborn Olfactory Bulb Interneurons in a Sensory Experience-Dependent Manner

**DOI:** 10.3389/fnins.2015.00514

**Published:** 2016-01-12

**Authors:** Sei-ichi Yoshihara, Hiroo Takahashi, Akio Tsuboi

**Affiliations:** Laboratory for the Molecular Biology of Neural Systems, Advanced Medical Research Center, Nara Medical UniversityKashihara, Japan

**Keywords:** adult neurogenesis, olfactory bulb interneuron, neural activity-dependent, dendritogenesis, spinogenesis, 5T4, Npas4

## Abstract

Inhibitory interneurons in the olfactory bulb are generated continuously throughout life in the subventricular zone and differentiate into periglomerular and granule cells. Neural circuits that undergo reorganization by newborn olfactory bulb interneurons are necessary for odor detection, odor discrimination, olfactory memory, and innate olfactory responses. Although sensory experience has been shown to regulate development in a variety of species and in various structures, including the retina, cortex, and hippocampus, little is known about how sensory experience regulates the dendritic development of newborn olfactory bulb interneurons. Recent studies revealed that the 5T4 oncofetal trophoblast glycoprotein and the neuronal Per/Arnt/Sim domain protein 4 (Npas4) transcription factor regulate dendritic branching and dendritic spine formation, respectively, in olfactory bulb interneurons. Here, we summarize the molecular mechanisms that underlie the sensory input-dependent development of newborn interneurons and the formation of functional neural circuitry in the olfactory bulb.

## Introduction

**Figure 1:**
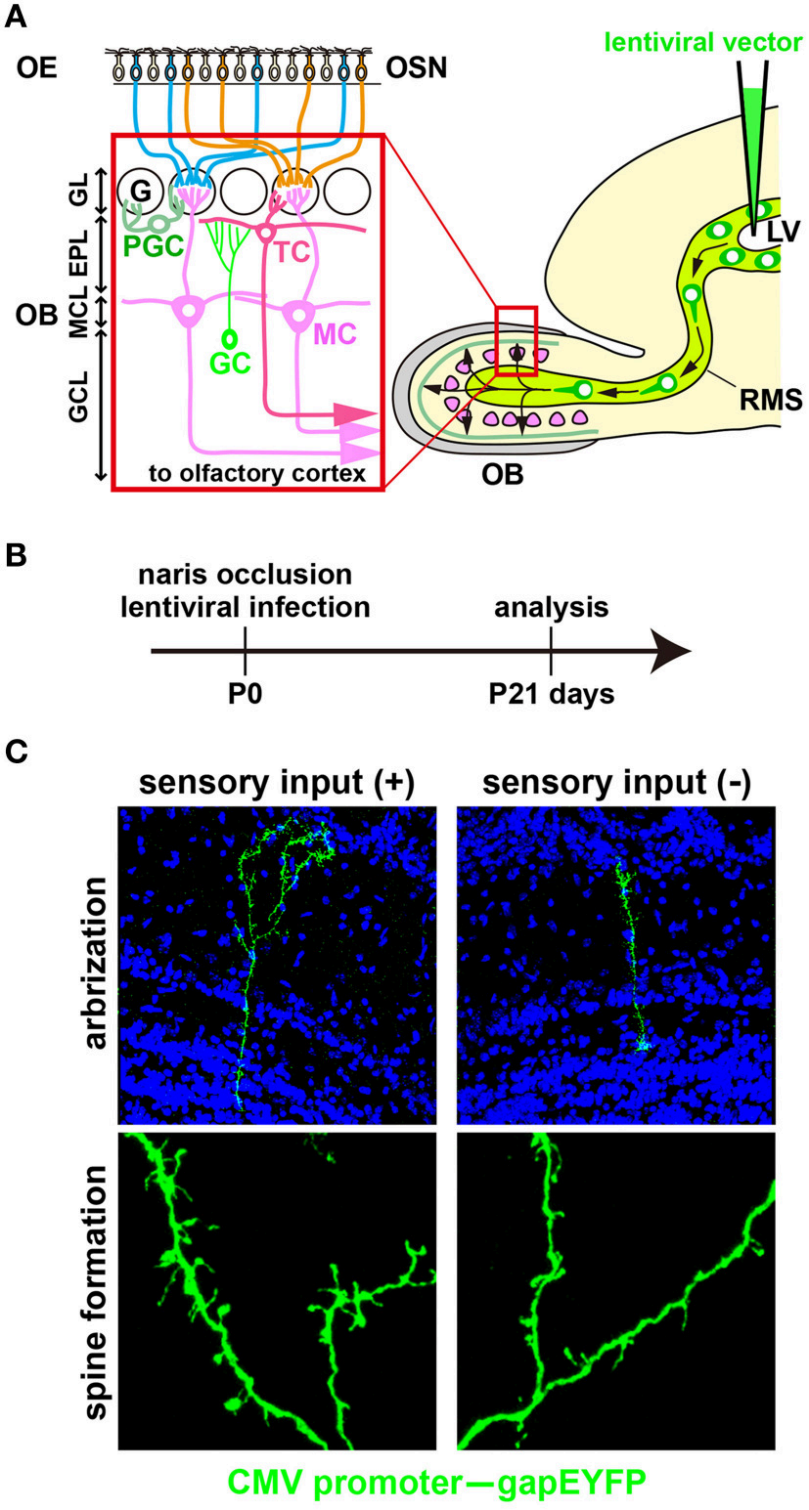
**Development of newborn OB interneurons is regulated by odor-evoked neural activity. (A)** Schematic representation of the olfactory bulb neural circuitry. Olfactory bulb (OB) interneurons are generated throughout life in the subventricular zone of the lateral ventricle (LV), migrate along the rostral migratory stream (RMS), and differentiate into γ-aminobutyric acid (GABA)-releasing inhibitory interneurons such as granule cells (GCs) and periglomerular cells (PGCs) in the OB. GCs and PGCs modulate the neural activity of excitatory projection neurons, including mitral and tufted cells (MC and TC) through dendrodendritic synapses between inhibitory and excitatory neurons. EPL, external plexiform layer; G, glomerulus; GCL, granule cell layer; GL, glomerular layer; MCL, mitral cell layer; OE, olfactory epithelium; OSN, olfactory sensory neuron. **(B)** A lentiviral vector (*CMV* promoter–*gapEYFP* gene) was injected into the lateral ventricle (LV) of wild-type mice at postnatal day 0 (P0). After 3 weeks (P21), YFP^+^ interneurons can be visualized in the olfactory bulb (OB). The activity-dependent development of newborn interneurons was analyzed by injecting a lentivirus into the LV of unilaterally naris-occluded mice. **(C)** Newborn granule cells (GCs) were analyzed in the open and closed sides of the OB from P21 naris-occluded mice. GC dendrites are less branched and have lower spine density in the closed than in the open side of the OB (modified from Yoshihara et al., 2012, 2014).

Sensory experience is recognized as a critical factor in the development and plastic modification of neural circuits in vertebrates (Katz and Shatz, 1996; Sanes and Lichtman, 2001; Nithianantharajah and Hannan, 2006; Lepousez et al., 2013). As well as newborn hippocampal neurons (Vadodaria and Jessberger, 2013), newborn olfactory bulb (OB) interneurons are a good model for studying the postnatal modification of neural circuits by sensory inputs from the external world. Specific odorants activate olfactory sensory neurons that express the corresponding odorant receptors (Mori and Sakano, 2011; Takeuchi and Sakano, 2014). The olfactory sensory neurons project their axons to specific glomeruli in the OB and can subsequently activate a specific neural circuit locally, facilitating the dendritic development of OB interneurons via interactions with excitatory projection neurons, such as mitral and tufted cells (Figure 1A; Mori and Sakano, 2011; Lepousez et al., 2013; Figueres-Oñate et al., 2014; Imai, 2014). Precursors for OB interneurons are generated throughout life in the subventricular zone of the lateral ventricle, migrate along the rostral migratory stream (RMS) and differentiate into γ-aminobutyric acid (GABA)-releasing inhibitory interneurons, such as periglomerular cells (PGCs) and granule cells (GCs; Figure 1A; Chazal et al., 2000; Alvarez-Buylla et al., 2008; Lledo et al., 2008; Whitman and Greer, 2009; Adam and Mizrahi, 2010; Kaneko et al., 2010; Sakamoto et al., 2011; Sequerra, 2014). Neural circuits that undergo reorganization by newborn OB interneurons are assumed to be essential for odor detection, odor discrimination, olfactory memory, and innate olfactory responses (Alonso et al., 2012; Sakamoto et al., 2014; Gschwend et al., 2015). It is well known that odor-evoked neural activity affects the survival and integration of newborn OB interneurons (Petreanu and Alvarez-Buylla, 2002; Rochefort et al., 2002; Yamaguchi and Mori, 2005; Bastien-Dionne et al., 2010; Lin et al., 2010; Sawada et al., 2011). In addition, elimination of GCs via cell death is promoted by top-down inputs from the olfactory cortex to the OB during the postprandial period (Yokoyama et al., 2011; Komano-Inoue et al., 2014). Moreover, odor deprivation and odor-enriched environments suppress and facilitate, respectively, dendritogenesis and spinogenesis in newborn OB interneurons (Saghatelyan et al., 2005; Kelsch et al., 2009; Livneh et al., 2009; Breton-Provencher et al., 2014; Lepousez et al., 2014). However, molecular mechanisms regulating the sensory experience-dependent dendritogenesis and spinogenesis in OB newborn interneurons remain unknown. Recent studies revealed that the 5T4 oncofetal trophoblast glycoprotein regulates the dendritic arborization of OB GCs in a sensory input-dependent manner (Yoshihara et al., 2012), whereas the neuronal Per/Arnt/Sim domain protein 4 (Npas4) transcription factor controls the sensory input-dependent dendritic spine formation of OB GCs (Yoshihara et al., 2014).

## Development of newborn OB interneurons is regulated by odor-induced neural activity

Neurogenesis arises continuously throughout life in two areas of the mouse brain, such as the subgranular zone of the dentate gyrus (Vadodaria and Gage, 2014) and the subventricular zone of the lateral ventricle (Tong and Alvarez-Buylla, 2014). In the latter, interneuronal neuroblasts migrate along the RMS to the OB (Figure 1A). After neuroblasts arrive at the OB, dendritogenesis and spinogenesis occur in those cells, which then differentiate into mature GABAergic inhibitory interneurons (GCs and PGCs) and incorporate into pre-existing neural circuits in the OB (Alvarez-Buylla et al., 2008; Lledo et al., 2008; Whitman and Greer, 2009; Adam and Mizrahi, 2010; Kaneko et al., 2010; Sakamoto et al., 2011; Sequerra, 2014). Previous studies showed that odor-evoked neural activity is required for the development of newborn OB interneurons at the following four steps.

*(1) Proliferation of neural stem cells and migration of neuroblasts*. An odor-enriched environment enhances proliferation of neural stem cells in both the RMS and subventricular zone, although chemical lesion of olfactory sensory neurons increases cell proliferation in the RMS alone (Alonso et al., 2008). When neuroblasts arrive at the OB, the direction of migration changes from rostral to radial (Hack et al., 2002; Saghatelyan et al., 2004; Belvindrah et al., 2011; Saha et al., 2012). Radial migration of neuroblasts in the OB is controlled by the secreted glycoprotein reelin (Hack et al., 2002) and the extracellular matrix glycoprotein tenascin-R (Saghatelyan et al., 2004; David et al., 2013). Because tenascin-R is produced by pre-existing GCs and expressed in a sensory input-dependent manner, its lack decreases the radial migration of neuroblasts as well as spine development of newborn GCs (Saghatelyan et al., 2004; David et al., 2013).

*(2) Differentiation, survival, and death of newborn interneurons*. When immature interneurons reach a given layer in the OB, they differentiate into mature PGCs and GCs. The production levels of the GABA synthetic enzyme (glutamic acid decarboxylase 67: GAD67) and GABA in OB interneurons are regulated in an activity-dependent manner (Parrish-Aungst et al., 2011; Lau and Murthy, 2012). A PGC subtype expresses *tyrosine hydroxylase* (*TH*) gene, encoding a rate-limiting enzyme for dopamine synthesis, in an odor input-dependent manner (Bastien-Dionne et al., 2010; Bovetti et al., 2013; Lazarini et al., 2014). The *TH* expression in PGCs is controlled by transcription factors such as Er81 (Cave et al., 2010) and COUP-TFI (Bovetti et al., 2013; Zhou et al., 2015) in an activity-dependent manner, whereas the transcription factor Pax6 is upregulated in TH-positive PGCs in odor-deprived mice (Bastien-Dionne et al., 2010). In unilaterally naris-occluded mice, the apoptotic rate of newborn GCs is increased on the closed side of the OB (Rochefort et al., 2002; Yamaguchi and Mori, 2005; Bastien-Dionne et al., 2010; Lin et al., 2010; Sawada et al., 2011), whereas their survival rate is increased in odor-enriched environments (Rochefort et al., 2002; Rochefort and Lledo, 2005). The survival and death of newborn PGCs are also regulated by sensory inputs. For example, newborn PGC death is induced by the connective tissue growth factor (CTGF) secreted from external tufted cells in the OB (Khodosevich et al., 2013). In odor-stimulated glomeruli, external tufted cells secrete more CTGF protein, enhancing death of newborn PGCs through transforming growth factor-β (TGF-β) receptor signaling downstream of CTGF (Khodosevich et al., 2013). In addition, olfactory deprivation negatively affects the survival of newborn calretinin-positive PGCs (Kato et al., 2012), whereas odor enrichment increases the cell number of TH-positive PGCs (Bonzano et al., 2014).

*(3) Dendritic morphogenesis of newborn interneurons*. In odor deprivation, the length and branching number of GC dendrites are decreased; by contrast, they are increased in an odor-enriched environment (Figures 1B,C; Saghatelyan et al., 2005; Yoshihara et al., 2012). GABA_A_ receptor mutant mice exhibit impaired dendritic branching and spine formation in OB GCs, suggesting that GABAergic synaptic transmission is important for proper dendritogenesis and spinogenesis (Pallotto et al., 2012). Furthermore, fragile X mental retardation protein (FMRP), which is an mRNA-binding protein essential for multiple aspects of neuronal mRNA metabolism, downregulates dendritic spine formation in OB GCs and is necessary for activity-dependent dendritic remodeling (Scotto-Lomassese et al., 2011).

*(4) Dendritic spine formation of newborn interneurons*. Through their dendritic spines, OB interneurons connect to projection neurons (mitral and tufted cells) to modulate activity (Lledo et al., 2008; Adam and Mizrahi, 2010; Imai, 2014). In odor-deprived mice, the dendritic spine formation of OB interneurons is suppressed in the distal dendritic domain and accelerated in the proximal dendritic domain; by contrast, dendritic spine formation is increased in the distal dendritic domain in odor-enriched environments (Figure 1C; Saghatelyan et al., 2005; Kelsch et al., 2009, 2012b; Livneh et al., 2009; Breton-Provencher et al., 2014; Lepousez et al., 2014). It is suggested that synapses of neonatal-born GCs retain a higher level of plasticity in response to changes in neural activity than those of adult-born GCs (Kelsch et al., 2012b). Odor input-dependent neural activity induces the formation and retraction of filopodia in the distal dendritic domain of GCs via NMDA receptor signaling (Kelsch et al., 2012a; Breton-Provencher et al., 2014). In OB GCs, odor-discrimination learning increases spine density in proximal dendritic domains, which receive top-down inputs from the olfactory cortex (Yokoyama et al., 2011; Komano-Inoue et al., 2014; Lepousez et al., 2014). Furthermore, corticotropin-releasing hormone (CRH) is produced in a subtype of OB interneurons at the external plexiform layer in an activity-dependent manner. When CRH is received by newly generated OB GCs, synaptogenesis in the GC dendrites is accelerated via CRH receptor signaling (Garcia et al., 2014).

## 5T4 glycoprotein regulates dendritic branching of newborn OB granule cells in a sensory input-dependent manner

Although odor-evoked neural activity is required for proper dendritic development of OB interneurons, its regulatory mechanisms remain unexplained. DNA microarray and *in situ* hybridization screenings in the unilaterally naris-occluded OB identified the oncofetal trophoblast glycoprotein gene, *5T4*, which is expressed in a specific subtype of OB interneurons following sensory experience (Imamura et al., 2006; Yoshihara et al., 2012). 5T4 is a type I membrane protein with an extracellular domain containing seven leucine-rich repeats bordered by characteristic leucine-rich repeat N- and C-flanking regions and a cytoplasmic domain containing a PDZ interaction motif (King et al., 1999). The 5T4 protein was first identified while searching for molecules with invasive properties shared by placental trophoblasts and cancer cells (Hole and Stern, 1990). The *5T4* gene expression is upregulated in many different carcinomas, while showing only low levels in most normal tissues (Southall et al., 1990) except for high levels in the brain and ovary (King et al., 1999; Barrow et al., 2005).

In the OB, the *5T4* gene is expressed not only in a subtype of PGCs at the glomerular layer, but also in a subtype of GCs (5T4-positive GCs) at the mitral-cell and superficial-GC layers (Imamura et al., 2006; Yoshihara et al., 2012). In the odor-deprived OB, the number of 5T4-positive GCs is decreased in the mitral-cell, and superficial-GC layers, indicating that the expression of *5T4* in 5T4-positive GCs is dependent on sensory inputs (Imamura et al., 2006; Yoshihara et al., 2012). Overexpression of *5T4* in newborn GCs by injecting lentiviral vectors into the lateral ventricle gives rise to more branched dendrites than those observed in control GCs (Yoshihara et al., 2012). In addition, *5T4*-overexpressing GCs have more branched dendrites even under sensory deprivation, whereas both *5T4* knockdown and knockout (KO) significantly reduce the dendritic branching of GCs in the OB (Yoshihara et al., 2012). Thus, 5T4 protein appears to be necessary for dendritic branching in OB interneurons.

**Figure 2:**
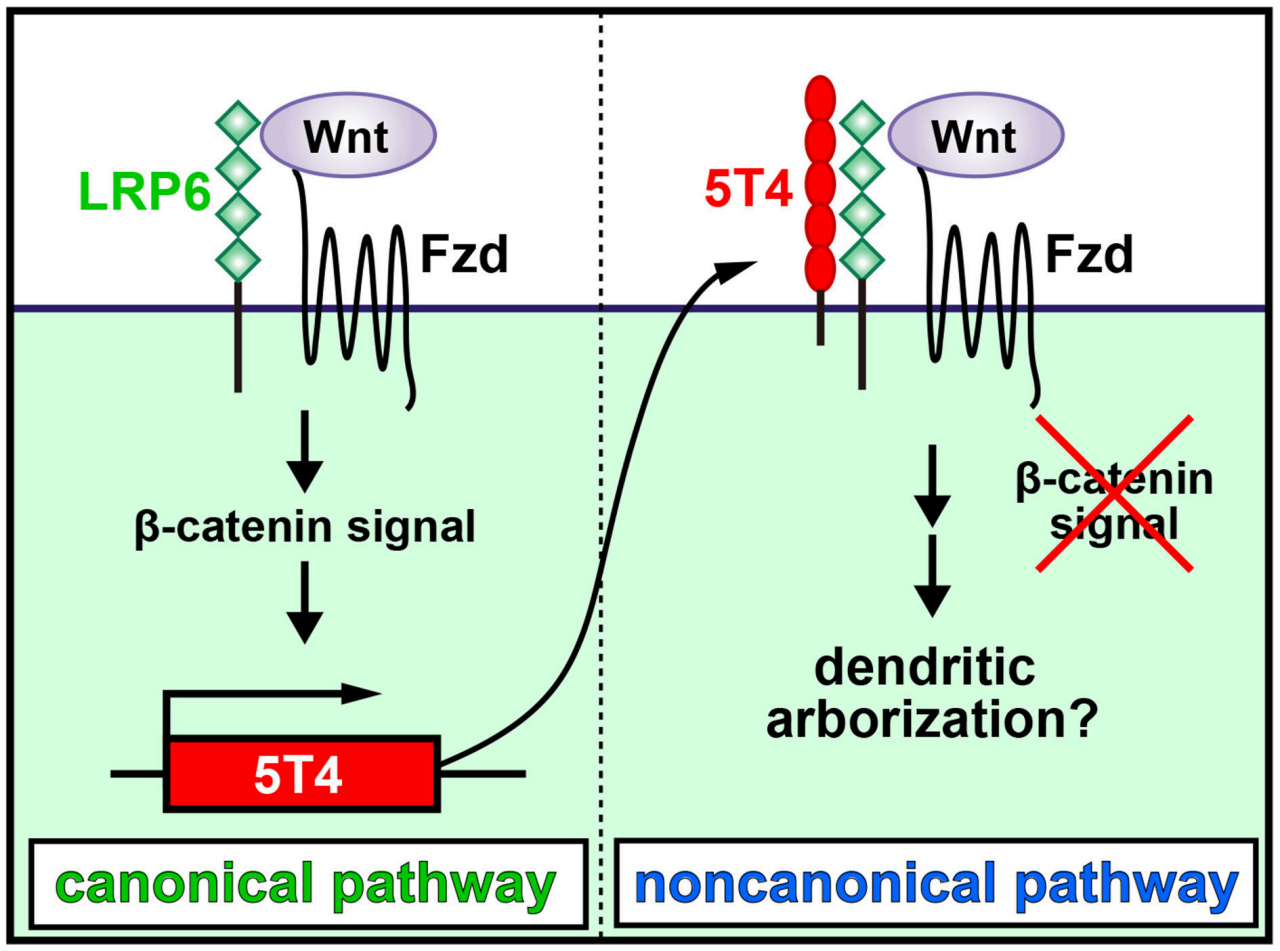
**Schematic diagram of dendritic development in newborn OB granule cells regulated by odor inputs**. 5T4 leucine-rich repeat (LRR)-containing transmembrane protein is induced by and downregulates the Wnt canonical pathway. Neural activity may induce the production of a canonical Wnt ligand to upregulate *5T4*, which subsequently blocks the canonical pathway and favors the noncanonical pathway in OB interneurons by inhibiting the internalization of a Wnt-coreceptor, low-density lipoprotein receptor-related protein 6 (LPR6). This leads to facilitation of dendritic arborization in OB GCs (modified from Kagermeier-Schenk et al., 2011; Yoshihara et al., 2012).

It was recently shown that 5T4 is both induced by and negatively regulates the Wnt canonical pathway, which then facilitates the response to the noncanonical pathway (Figure 2; Kagermeier-Schenk et al., 2011; Zhao et al., 2014). Thus, neural activity may induce the production of a canonical Wnt ligand to upregulate *5T4*, which subsequently blocks the canonical pathway and favors the noncanonical pathway in OB interneurons by inhibiting the internalization of a Wnt-coreceptor, low-density lipoprotein receptor-related protein 6 (LPR6) (Kagermeier-Schenk et al., 2011; Zhao et al., 2014). In fact, compared with the wild type, disruption of the *Wnt5a* gene, which encodes a noncanonical Wnt ligand expressed in a subtype of OB interneurons, gives rise to reduced dendritic extension in GCs (Pino et al., 2011). It is possible that Wnt5a production regulates the noncanonical planar cell polarity pathway, leading to facilitation of dendritic arborization (van Amerongen and Nusse, 2009; Hirota et al., 2012). Recently, *5T4* deletion and domain-swap experiments established that the 5T4 intracellular domain without a PDZ-binding motif is necessary and sufficient for the dendritic branching of OB GCs, but the 5T4 extracellular leucine-rich repeat domain is not necessary for dendritic branching (Yoshihara et al., 2012). However, the 5T4 extracellular domain is reportedly essential for inhibition of Wnt/β-catenin signaling (Kagermeier-Schenk et al., 2011; Zhao et al., 2014). Therefore, it is likely that 5T4 regulates dendritic branching in a Wnt signaling-independent manner. Because the 5T4 intracellular region may interact with cytoskeletal proteins to regulate the dendritic arborization of GCs, the identification of 5T4-associating proteins will enable us to understand the mechanisms regulating the dendritic development of OB interneurons in an activity-dependent manner.

## Npas4 transcription factor regulates dendritic spine formation of newborn OB granule cells in a sensory input-dependent manner

Although odor-induced neural activity is required for spine formation in OB interneurons, its regulatory mechanism remains unresolved. DNA microarray and *in situ* hybridization screenings identified a transcription factor gene, *Npas4*, which is expressed in a subtype of OB GCs following sensory experience (Bepari et al., 2012; Yoshihara et al., 2014). *Npas4* is an immediate early gene induced by neural activity via a calcium-dependent signaling pathway (Lin et al., 2008; Pruunsild et al., 2011; Ramamoorthi et al., 2011; Bloodgood et al., 2013). In addition, Npas4 promotes the formation of inhibitory synapses in the developing visual system (Lin et al., 2008; Bloodgood et al., 2013) and adjusts the homeostatic inhibitory/excitatory balance in excitatory neurons to induce visual cortical plasticity (Maya-Vetencourt et al., 2012). Moreover, the Npas4 protein interacts with several promoters regulated by neural activity and mediates *brain-derived neurotrophic factor* (*BDNF*) gene expression in cortical pyramidal and hippocampal CA3 neurons (Lin et al., 2008; Pruunsild et al., 2011; Ramamoorthi et al., 2011; Bloodgood et al., 2013).

Overexpression of *Npas4* in newborn OB GCs by injecting lentiviruses into the lateral ventricle gives rise to an increase in spine density even under sensory deprivation conditions (Yoshihara et al., 2014). Furthermore, *Npas4* overexpression increases the number of puncta stained by either the postsynaptic marker gephyrin or pre-synaptic marker synaptoporin at the distal region of GC dendrites. By contrast, both *Npas4* knockdown and KO cause a significant reduction in the spine density of GC dendrites (Yoshihara et al., 2014). Thus, Npas4 is necessary and sufficient for increasing sensory input-dependent synaptogenesis in OB GCs. Interestingly, Npas4 is also required for activity-dependent spine development of adult-born GCs in the hippocampal dentate gyrus (Sim et al., 2013).

**Figure 3:**
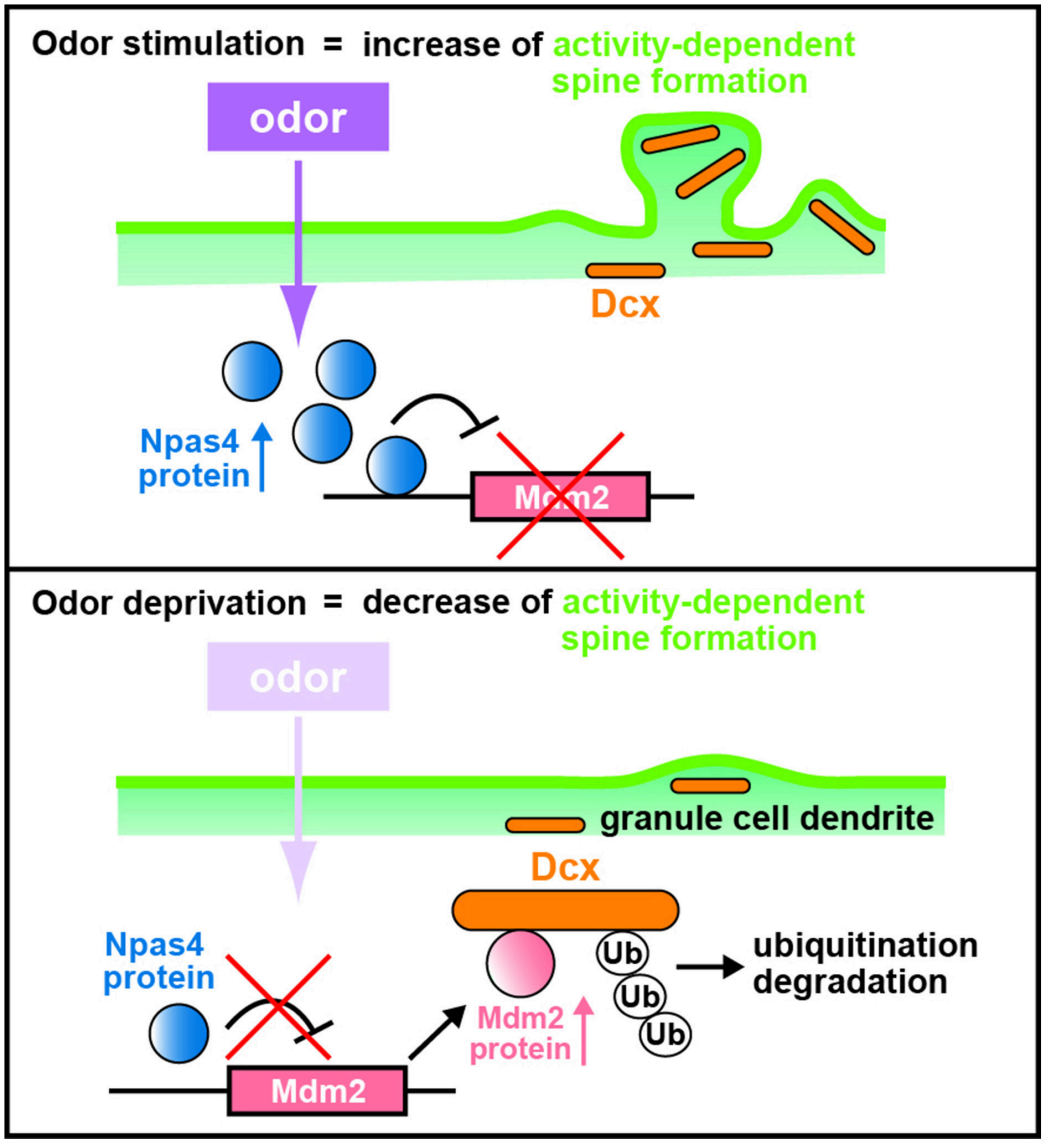
**Schematic diagram of dendritic spine formation in newborn OB granule cells regulated by odor inputs**. In wild-type olfactory bulb (OB) granule cells (GCs), odor stimulation induces *Npas4* expression to suppress *Mdm2* expression. The suppression of *Mdm2* promotes the spine formation of GC dendrites mediated by doublecortin (Dcx). By contrast, odor deprivation decreases *Npas4* expression in OB GCs to upregulate the expression of *Mdm2*, leading to ubiquitination of Dcx. This results in degradation of Dcx and reduced dendritic spine density in OB GCs, as in the case of *Npas4*-knockout OB GCs (modified from Yoshihara et al., 2014).

The mechanism for the Npas4 regulation of synaptogenesis in OB interneurons was explored using chromatin immunoprecipitation sequencing (ChIP-Seq) to search for Npas4 target genes in homogenized OB tissues with an Npas4 antibody associating with the promoter regions that bind Npas4. A novel target of Npas4, the oncogenic E3 ubiquitin ligase gene, *murine double minute 2* (*Mdm2*), is expressed at low levels in the wild-type OB but at higher levels in the *Npas4* KO OB (Yoshihara et al., 2014). Lateral ventricle injections of lentiviruses to either overexpress or knockdown *Mdm2* showed reduction and enhancement, respectively, in the spine density of GC dendrites compared with those in controls (Yoshihara et al., 2014), demonstrating that *Mdm2* is a *bona fide* target gene of Npas4 and that *Mdm2* expression is suppressed by Npas4 (Figure 3).

Mdm2 is localized at synapses, ubiquitinates and degrades postsynaptic density protein-95 (PSD-95) in rat hippocampal neurons (Colledge et al., 2003). However, according to the data of western blot analysis, the amount of PSD-95 protein is similar in wild-type and *Npas4* KO OBs (Yoshihara et al., 2014). Thus, to reveal the mechanism by which Mdm2 regulates synaptogenesis in OB interneurons, Mdm2 target proteins that are produced differentially between wild-type and *Npas4* KO OBs were searched using isobaric tags for relative and absolute quantitation (iTRAQ) proteomics (Yoshihara et al., 2014). Proteomic and cell-line analyses revealed that Mdm2 ubiquitinates and leads to the degradation of a microtubule-associated protein, doublecortin (Dcx). Dcx is generally used as a marker for immature neurons in the adult neurogenic lineage (Brown et al., 2003; Saaltink et al., 2012). Dcx regulates the migration and dendritic development of migrating neurons in the OB core region, including the RMS and the deep GC layer (Ocbina et al., 2006; Belvindrah et al., 2011). Immunohistochemical analysis of OB sections with a Dcx antibody indicates that the intensity of immunofluorescent signals in GC dendrites at the external-plexiform and GC layers are two-fold lower in the *Npas4* KO OB than in the wild-type OB (Yoshihara et al., 2014). Furthermore, overexpression and knockdown of *Dcx* achieved by injecting lentiviruses into the lateral ventricle enhance and reduce, respectively, spine density in GC dendrites (Yoshihara et al., 2014). Thus, Dcx plays an important role in increasing the dendritic spine density of OB GCs: Npas4 protein inhibits *Mdm2* expression, which prevents ubiquitination and degradation of Dcx, thereby promoting dendritic spine development in OB GCs following sensory experience (Figure 3).

Interestingly, two Dcx homologs, Dcx-like kinases 1 and 2 (Dclk1/Dclk2), reportedly regulate dendritic spine formation in hippocampal neurons (Shin et al., 2013). It was also recently demonstrated that Dcx is necessary for synapse formation in proper neuromuscular junction in the mouse and human (Bourgeois et al., 2015). Dcx and Dclk1/Dclk2 bind to an actin-binding protein, spinophilin, which is known to regulate spine morphology (Tsukada et al., 2003). Dcx also induces the bundling and cross-linking of microtubules and F-actin (Tsukada et al., 2005). Thus, the microtubule-binding protein Dcx family may play a crucial role in dendritic spine development of OB GCs.

## Perspectives

In the cerebral cortex and hippocampus, neural activity regulates a program of gene transcription to affect synaptic development and plasticity (Ebert and Greenberg, 2013). Elevation of intracellular calcium levels induced by neural activity leads to activation of genes for multiple signaling molecules, including calmodulin kinase II (CaMK II), protein kinase A, cyclic AMP-responsive element-binding protein (CREB), and calcineurin (Ebert and Greenberg, 2013; Kawashima et al., 2014). CREB signaling is required for the survival, migration, and dendritic development of OB interneurons (Herold et al., 2011). Activation of the multiple signaling cascades facilitates the expression of neural activity-dependent genes, including the immediate early genes *c-fos, egr1*, and *Arc* (Ebert and Greenberg, 2013; Kawashima et al., 2014). A recent study found that neuronal activity induces DNA breaks in the promoters of immediate early genes and facilitates their expression (Madabhushi et al., 2015). Among the immediate early genes, c-fos and egr1 transcription factors activate genes for BDNF and Arc to regulate synaptic development (Ebert and Greenberg, 2013). In newborn OB interneurons, several immediate early genes (*c-fos, egr1*, and *Arc*) are expressed in a sensory input-dependent manner (Guthrie et al., 1993; Inaki et al., 2002; Busto et al., 2009; Bepari et al., 2012). Because the expression of the *5T4* gene is at a basal level in OB GCs after exiting the RMS, it is important to explore how the odor input-dependent expression is regulated by transcription factors, including those immediate early genes described above. It was recently reported that *BDNF* overexpression increases dendritic spine density of GCs (McDole et al., 2015). Therefore, future studies that identify the target genes of these immediate early genes, including *Npas4*, will allow us to understand the molecular mechanisms underlying activity-dependent development in newborn OB interneurons.

The functional significance of sensory input-dependent development in newborn OB interneurons has been explored. Olfactory experiences, such as odor-enrichment and odor-discrimination learning, regulate the maturation and survival of adult-born OB interneurons (Alonso et al., 2006; Livneh et al., 2009; Breton-Provencher et al., 2014; Lepousez et al., 2014). Newborn OB interneurons possess specific properties that are different from those of pre-existing interneurons, such as enhanced synaptic plasticity during a critical time window (Carleton et al., 2003; Panzanelli et al., 2009; Pallotto et al., 2012; Livneh et al., 2014), suggesting that these newborn interneurons uniquely contribute to odor processing. Consistent with this suggestion, odor detection and odor-discrimination learning are reportedly impaired in mice with diminished adult neurogenesis in the OB (Gheusi et al., 2000; Enwere et al., 2004; Breton-Provencher et al., 2009). However, the genetic ablation of adult-born interneurons causes deficits in innate olfactory responses, including predator avoidance and sexual behaviors (Sakamoto et al., 2011), but not in other normal olfactory abilities, such as odor detection and simple odor discrimination (Imayoshi et al., 2008; Sakamoto et al., 2011). This incongruence could be attributable to differences in the subtypes and numbers of OB interneurons that were manipulated in the individual studies. However, none of the methodologies used in these studies, including physically or genetically eliminating newborn cells, block the birth of adult-born OB interneurons in a spatially and temporally specific manner (Gheusi et al., 2000; Enwere et al., 2004; Imayoshi et al., 2008; Breton-Provencher et al., 2009; Sultan et al., 2010; Lazarini et al., 2014). A genetic activation study in which channelrhodopsin-2 was selectively expressed in newborn GCs showed that photostimulation of adult-born neurons (2 months old) facilitates difficult odor-discrimination learning and improves odor memory, whereas photostimulation of postnatal day 6-born neurons does not (Alonso et al., 2012; Gschwend et al., 2015). On the other hand, genetic inhibition of synaptic transmission in postnatal-born neurons impairs difficult odor-discrimination learning (Sakamoto et al., 2014). Because Dcx is produced in younger mature GCs, but is absent in older mature GCs (Brown et al., 2003), Npas4 may regulate dendritic spine formation in the younger but not the older mature GCs (Yoshihara et al., 2014). Interestingly, conditional KO of *Npas4* function in OB neurons impairs difficult odor-discrimination learning without affecting the ability to detect odors (Yoshihara et al., 2014), suggesting that spine development in newborn younger GCs is required in part for olfactory behaviors. By regulating the activity-dependent synaptic development of newborn OB GCs, Npas4 may play a role in shaping the functional neural circuitry involved in olfactory discrimination learning. Collectively, these studies provide important evidence that newborn OB interneurons, in which spine formation is regulated by Npas4 in a sensory experience-dependent manner, play a functional role in the OB circuitry and thus in its behavioral manifestation. Because GCs can be divided into several subtypes (Merkle et al., 2014), it is assumed that each GC subtype forms a distinct local circuit in the OB (Mori et al., 1983; Orona et al., 1983; Shepherd et al., 2004). Thus, future studies that individually manipulate the subtypes of OB interneurons in a spatially and temporally specific manner will help us to understand their functions in the OB circuitry.

## Author contributions

SY, HT, and AT wrote the paper.

## Funding

This work was supported by Grants-in-Aid for Scientific Research on (B) (AT), (C) (HT and SY), and Innovative Areas (Adaptive circuit shift) (AT) from the Ministry of Education, Culture, Sports, Science and Technology (MEXT), Japan. AT was supported by grants from the Smoking Research Foundation, Japanese Applied Enzymology Foundation, Senshin Medical Research Foundation, and Ono Medical Research Foundation, and a Nara Medical University Grant-in-Aid for Collaborative Research Projects in Japan. SY and HT were supported by grants from Takeda Science Foundation and Astellas Foundation for Research on Metabolic Disorders, Japan. SY was supported by a grant from Terumo Foundation for Life Sciences and Arts. HT was supported by grants from the Salt Science Research Foundation (no. 14C3), Mishima Kaiun Memorial Foundation, and Banyu Life Science International Foundation in Japan.

### Conflict of interest statement

The authors declare that the research was conducted in the absence of any commercial or financial relationships that could be construed as a potential conflict of interest.
